# Immunogenic cell death-related risk signature for tumor microenvironment profiling and prognostic prediction in colorectal cancer

**DOI:** 10.17305/bb.2025.12028

**Published:** 2025-04-17

**Authors:** Pengcheng Wang, Wei Zhao, Linghong Guo, Hailei Cao

**Affiliations:** 1 Colorectal Surgery, Shanxi Province Cancer Hospital/Shanxi Hospital Affiliated to Cancer Hospital, Chinese Academy of Medical Sciences/Cancer Hospital Affiliated to Shanxi Medical University, Taiyuan, China; 2 Department of Anesthesiology, The First Affiliated Hospital of Harbin Medical University, Harbin, China; 3 Department of Colorectal and Anal Surgery, Shanxi Province Cancer Hospital/Shanxi Hospital Affiliated to Cancer Hospital, Chinese Academy of Medical Sciences/Cancer Hospital Affiliated to Shanxi Medical University, Taiyuan, China

**Keywords:** Colorectal cancer, CRC, drug resistance, prognostic signature, immunogenic cell death, ICD, tumor immune microenvironment, TIME, cancer

## Abstract

Immunogenic cell death (ICD) reshapes the tumor immune microenvironment and activates the adaptive immune response. However, the clinical significance of ICD-associated genes in colorectal cancer (CRC) remains unclear. In this study, we used weighted gene co-expression network analysis (WGCNA) to identify ICD-related gene modules. An ICD-related risk score (ICDRS) was then constructed using Cox regression modeling and LASSO analysis. Immune cell infiltration in patients with different risk levels was assessed using the ESTIMATE and single-sample Gene Set Enrichment Analysis algorithms (GSEA). The oncoPredict package was employed to explore the association between the ICDRS and chemotherapy drug sensitivity. Finally, the expression levels of ICD-related genes were validated through *in vitro* cellular experiments. Three CRC prognostic genes—*CLMP*, Neuropilin-1 (*NRP1*), and *PLEKHO1*—were identified from a set of 34 ICD-associated genes based on WGCNA and LASSO analyses. These genes were used to construct the ICDRS model. Notably, a high ICDRS was found to be an independent predictor of poorer overall survival (OS) in CRC patients. High-risk patients also exhibited increased immune cell infiltration. Moreover, the ICDRS was significantly correlated with sensitivity to conventional chemotherapeutic drugs, suggesting its potential utility in guiding personalized chemotherapy. Cellular assays confirmed that *CLMP*, *NRP1*, and *PLEKHO1* were differentially expressed between normal and cancerous cells, and that *NRP1* specifically promoted the proliferation, migration, and invasion of CRC cells. In conclusion, the ICDRS may serve as a reliable predictor of CRC prognosis and offers a promising direction for the clinical management of CRC patients.

## Introduction

Colorectal cancer (CRC) ranks as the third most frequently diagnosed cancer worldwide. Its annual incidence has reached 1.9 million cases, accounting for approximately 10% of all newly diagnosed cancers globally. Notably, there is a rapid rise in CRC incidence among younger populations in both developed and developing regions [1, 2]. The progression of CRC is a complex, multistep process involving various genetic alterations. CRC cells display distinct biological behaviors, including aggressive proliferation, a high tendency for relapse, and the potential to metastasize [3, 4]. Despite advances in treatment, CRC survival rates remain poor [5–7], highlighting the urgent need for novel prognostic, therapeutic, and diagnostic biomarkers.

Immunogenic cell death (ICD) is a form of cell death [8, 9] characterized by an active interaction between immune cells and dying cells, representing a key mode of communication between the immune system and tumor cells [10]. ICD primarily occurs through apoptosis, during which damage-associated molecular patterns (DAMPs) are released from tumor cells. These DAMPs are recognized by NOD-like receptors (NLRs) and innate immune receptors such as Toll-like receptors (TLRs), triggering immune responses that specifically target tumor cells. This dual mechanism—directly killing cancer cells while enhancing antitumor immunity—can both promote and prolong the effectiveness of chemotherapeutic drugs [11, 12]. A previous study developed and validated an ICD risk signature for lower-grade glioma based on the expression, function, and genetic alterations of 34 ICD-associated genes, ultimately identifying a 12-gene signature [13]. Additionally, two ICD-related subtypes were identified using consensus clustering, and an ICD-associated prognostic model was established to predict survival in patients with head and neck squamous cell carcinoma [14]. In another study, single-cell analysis of ascending thoracic aortic aneurysms revealed that endothelial cells were the primary targets of ICD. In this context, the aortic endothelial cell receptor ACKR1 promoted the infiltration of T cells and myeloid cells through interaction with CCL5 and CXCL8 ligands, respectively [15]. These findings suggest that identifying effective ICD-related biomarkers may improve clinical outcomes for patients with CRC. The role of the immune system in cancer initiation, progression, and treatment has been extensively studied. Recent therapeutic research underscores the importance of the interaction between dying or dead cancer cells and immune cells in determining the efficacy of cancer therapies [16]. ICD stimulates both innate and adaptive immune responses, contributing to the development of long-lasting immunological memory [17–19]. Similarly, many cancer treatments aim to induce ICD to enhance antitumor immunity and establish durable immune protection against cancer recurrence [20].

The aim of this study was to construct a risk score model based on ICD-related genes to evaluate its potential application in prognosis prediction, tumor immune microenvironment characterization, and personalized treatment guidance for patients with CRC. An ICD-related risk score (ICDRS) was established using weighted gene co-expression network analysis (WGCNA) to identify genes correlated with ICD in CRC. The prognostic value and independent predictive performance of the ICDRS were subsequently validated. Additionally, the ICDRS was analyzed in relation to somatic mutation status and copy number alterations (CNAs) through molecular characterization. Functional pathway alterations and immune cell infiltration patterns were also assessed. In conclusion, the ICDRS model demonstrates potential as an independent prognostic indicator for CRC and may offer novel biomarkers and therapeutic targets to support precision immunotherapy and personalized chemotherapy strategies.

## Materials and methods

### Data acquisition and preprocessing

Bulk-sequencing data in the form of FPKM values were log2-transformed. Survival data for 367 primary CRC samples and 51 normal samples from The Cancer Genome Atlas Program (TCGA, https://cancergenome.nih.gov) were processed using the R package TCGAbiolinks [21] and used as the training cohort (TCGA-COADREAD). Somatic mutation data (MAF files) and CNA data based on whole-exome sequencing were also obtained from the TCGA database. For validation, clinical data and gene expression profiles of 50 CRC patients were collected from the Gene Expression Omnibus (GEO; accession number: GSE17537; https://www.ncbi.nlm.nih.gov/geo/). Clinical characteristics of patients from both the TCGA-COADREAD and GSE17537 datasets are summarized in Table S1. For genes with multiple probes, the median expression value was used to represent gene expression.

### WGCNA analysis and key ICD-associated genes

ICD-related genes identified in a previous study [22] were used to calculate ICD enrichment scores for each sample using the R package GSVA [23]. Co-expression network analysis was conducted with the R package WGCNA [24], applying a soft-thresholding power of three, which yielded a scale-free topology fit index of 0.85. Samples with the highest ICD enrichment scores and the top 50% median absolute deviation (MAD) in expression profiles were included in the co-expression network analysis. Modules containing at least 30 genes were identified through hierarchical clustering. ICD-related modules were selected based on their correlation with clinical data, with the pink and turquoise modules chosen for further analysis. Genes within these modules that exhibited high module membership (MM > 0.8) and gene significance (GS > 0.6) were considered as hub genes.

### Construction and evaluation of the ICDRS

Based on the expression value of the selected ICD-related modules, prognostic markers were identified using univariate Cox proportional hazard regressions (*P* values < 0.05). Next, the ICDRS was developed with LASSO-penalized Cox regression. The LASSO penalty parameter λ was refined to determine the coefficient for each gene, and the ICDRS was formulated as follows: (1)
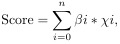
where χi represented the expression of a gene and *βi* represented the gene’s coefficient from the LASSO-penalized Cox regression model. Low-risk and high-risk patients were accordingly grouped by the median ICDRS value.

### The correlation between clinical features and the ICDRS

Univariate Cox regression analysis was conducted to evaluate the association between clinical factors—such as gender, age, TNM stage, lymphatic invasion, and risk scores—and patient survival [25]. The independent prognostic value of the ICDRS was further assessed using multivariate Cox proportional hazards regression. Differences in clinical characteristics between the two risk groups were compared using the Wilcoxon rank-sum test.

### Analyses of functional and pathway enrichment

Gene Ontology (GO) and Kyoto Encyclopedia of Genes and Genomes (KEGG) enrichment analyses were performed using the R package clusterProfiler [26, 27]. An FDR-adjusted *P* value < 0.05 was considered statistically significant. To identify highly enriched gene sets (nominal *P* values <0.05 and FDR-adjusted *P* values < 0.05), gene set variation analysis (GSVA) was conducted using the 50 hallmark gene sets from the MSigDB database.

### Estimation of immune cell infiltration

Immune infiltration in each sample was assessed using the single-sample Gene Set Enrichment Analysis (ssGSEA) algorithm, based on the expression levels of immune cell-specific markers [28]. This method was chosen because ssGSEA does not depend on a reference dataset, making it particularly suitable for RNA-seq data and allowing for a comprehensive evaluation of immune cell infiltration at the individual sample level. The resulting immune infiltration scores were analyzed to investigate their correlation with the ICDRS and their potential role in the tumor immune microenvironment of CRC. Additionally, tumor purity, as well as the abundance of intratumoral, stromal, and immune cells within the tumor microenvironment (TME), were estimated using the ESTIMATE algorithm based on the gene expression profiles of CRC tissues [29].

### Genetic variation analysis

Genetic variation analysis was conducted based on single nucleotide polymorphisms (SNPs) and copy number variations (CNVs) obtained from the TCGA database. Mutation types and gene mutation frequencies were visualized using the R package maftools [30]. CNA summary plots were generated with the ggplot2 package to illustrate chromosomal changes. Additionally, Circos plots were created using the RCircos package [31] to display the genomic distribution of ICD-correlated genes.

### Drug sensitivity determination

The GDSC v2 database (http://www.cancerrxgene.org) provides gene expression and drug response data for cancer cell lines, enabling correlation analysis between drug sensitivity and risk scores. Drug response prediction was performed using the R package oncoPredict [32, 33]. The half-maximal inhibitory concentration (IC50) represents the drug concentration required to achieve 50% of its maximal inhibitory effect, with lower IC50 values indicating higher sensitivity. The association between chemotherapy sensitivity and risk scores was evaluated using Spearman correlation analysis.

### Cell culture and cell transfection

DMEM medium containing 1% antibiotic/antifungal solution and 10% fetal bovine serum (FBS) was used to culture the Caco2 (CRC cell line) and NCM460 (normal colonic mucosal epithelial cell line) purchased from the American Type Culture Collection (ATCC) at 37 ^∘^C with 5% CO_2_. Following the manufacturer’s guidelines, Lipofectamine 2000 (Invitrogen, Carlsbad, CA, USA) was utilized for cell transfection. Briefly, Caco2 cells were seeded at a density of 2 × 10^5^ cells per well in a six-well plate and transfected with siRNA at a final concentration of 50 nM using 5 µL of Lipofectamine 2000 per well. To downregulate the Neuropilin-1 (*NRP1*) gene, Caco2 cells were transfected with *NRP1*-specific siRNA (si-*NRP1*#1: 5′-CAGCCTTGAATGCACTTATAT-3′ and si-*NRP1*#2: 5′-CAGAAGAATGGTACAAATCCAAG-3′, Sigma-Aldrich, St. Louis, MO, USA), while the controls were transfected with the corresponding non-specific control siRNA (si-NC, Sigma-Aldrich, St. Louis, MO, USA). After the transfection, the cells were cultured in an incubator for 48 h for subsequent experimental analysis.

### Quantitative reverse transcriptase PCR (qRT-PCR)

Following the manufacturer’s guidelines, TRIzol reagent (Invitrogen, Carlsbad, CA, USA) was employed to separate total RNA, which was reverse-transcribed into cDNA using the PrimeScript RT kit (Takara Bio, Shiga, Japan). To quantify the expression levels of the *CLMP*, *PLEKHO*, and *NRP1* genes, qRT-PCR analysis was performed with the use of SYBR Green PCR Master Mix (Applied Biosystems, Foster City, CA, USA) on an ABI 7500 real-time PCR system (Applied Biosystems, Foster City, CA, USA), strictly following the instructions. The primer sequences were listed as follows: *CLMP* Forward Sequence 5′-3′: TCCTACTATGTTGGAACCTTGGG and Reverse Sequence 5′-3′: CGGTGAGCAGCCATTCAATATC; *PLEKHO1* Forward Sequence 5′-3′: GGGACCAGCTCTACATCTCTG and Reverse Sequence 5′-3′: TGGAGTGGGCAAGAGTAAACT; *NRP1* Forward Sequence 5′-3′: GGCGCTTTTCGCAACGATAAA and Reverse Sequence 5′-3′: TCGCATTTTTCACTTGGGTGAT. *GAPDH* Forward Sequence 5′-3′: GTCTCCTCTGACTTCAACAGCG and Reverse Sequence 5′-3′: ACCACCCTGTTGCTGTAGCCAA.

### CCK-8 assay

Caco-2 cells in the logarithmic growth phase were seeded into a 96-well plate at a density of 1 × 10^4^ cells per well and incubated at 37 ^∘^C with 5% CO_2_ for 0, 24, 48, or 72 h. Following incubation, 10 µL of CCK-8 solution was added to each well, and the plate was further incubated at 37 ^∘^C for 2 h. Absorbance at 450 nm was then measured to generate the CCK-8 curve, with absorbance values plotted on the *Y*-axis and time on the *X*-axis.

### Wound healing test

A total of 4 × 10^5^ Caco-2 cells were suspended in 10 mL of medium and seeded into a 10-cm dish. Once the cells reached 95% confluency, uniform wounds were created in the cell monolayer using the tip of a 100 µL pipette. The scratches were then washed with PBS and the remaining cells were incubated in complete medium containing 1% FBS at 37 ^∘^C in 5% CO_2_. Scratch width was observed under an inverted microscope at 0 and 48 h post-wounding. Images were analyzed using ImageJ software (version 1.51n).

### Transwell assay

A cell invasion assay was performed using Matrigel (BD Biosciences, San Jose, CA, USA) to pre-coat the upper chambers of Transwell inserts (8.0 µm pore size, Corning Inc., Corning, NY, USA). Transfected Caco-2 cells (si-NRP1 and si-NC) were suspended in FBS-free DMEM and seeded into the upper chambers, while DMEM containing 20% FBS was added to the lower chambers as a chemoattractant. After 24 h of incubation, non-invading cells on the upper surface were removed, and cells that had invaded through the membrane were fixed with 4% formaldehyde and stained with 0.1% crystal violet. Invaded cells were counted under a microscope (Olympus Corporation, Tokyo, Japan).

### Statistical analysis

All statistical analyses were performed using the R language (https://www.R-project.org) or GraphPad Prism 8.0.2 (GraphPad Inc., La Jolla, CA, USA). Prior to hypothesis testing, the normality of data distribution was assessed using the Shapiro–Wilk test. For normally distributed data, results were presented as mean ± standard deviation (SD), while for non-normally distributed data, results were expressed as median with interquartile range. Continuous variables between groups were compared using the Wilcoxon rank-sum test for non-normally distributed data and the Student’s *t*-test for normally distributed data. The log-rank test was used to determine statistically significant differences in survival durations between groups under investigation. Independent prognostic factors associated with survival were identified using univariate and multivariate Cox proportional hazards regression analysis. ICD-related gene associations were assessed by Spearman correlation analysis. Unpaired *t*-tests, one-way analysis of variance, and two-way analysis of variance were applied during the statistical analysis of experimental data.

Statistical significance was set at *P* values < 0.05. ns: not significant (*P* values > 0.05); ^*^P values < 0.05; ^**^P values < 0.01; ^***^P values < 0.001; ^****^P values <0.0001.

## Results

### ICD-related gene changes in CRC

**Figure 1.**
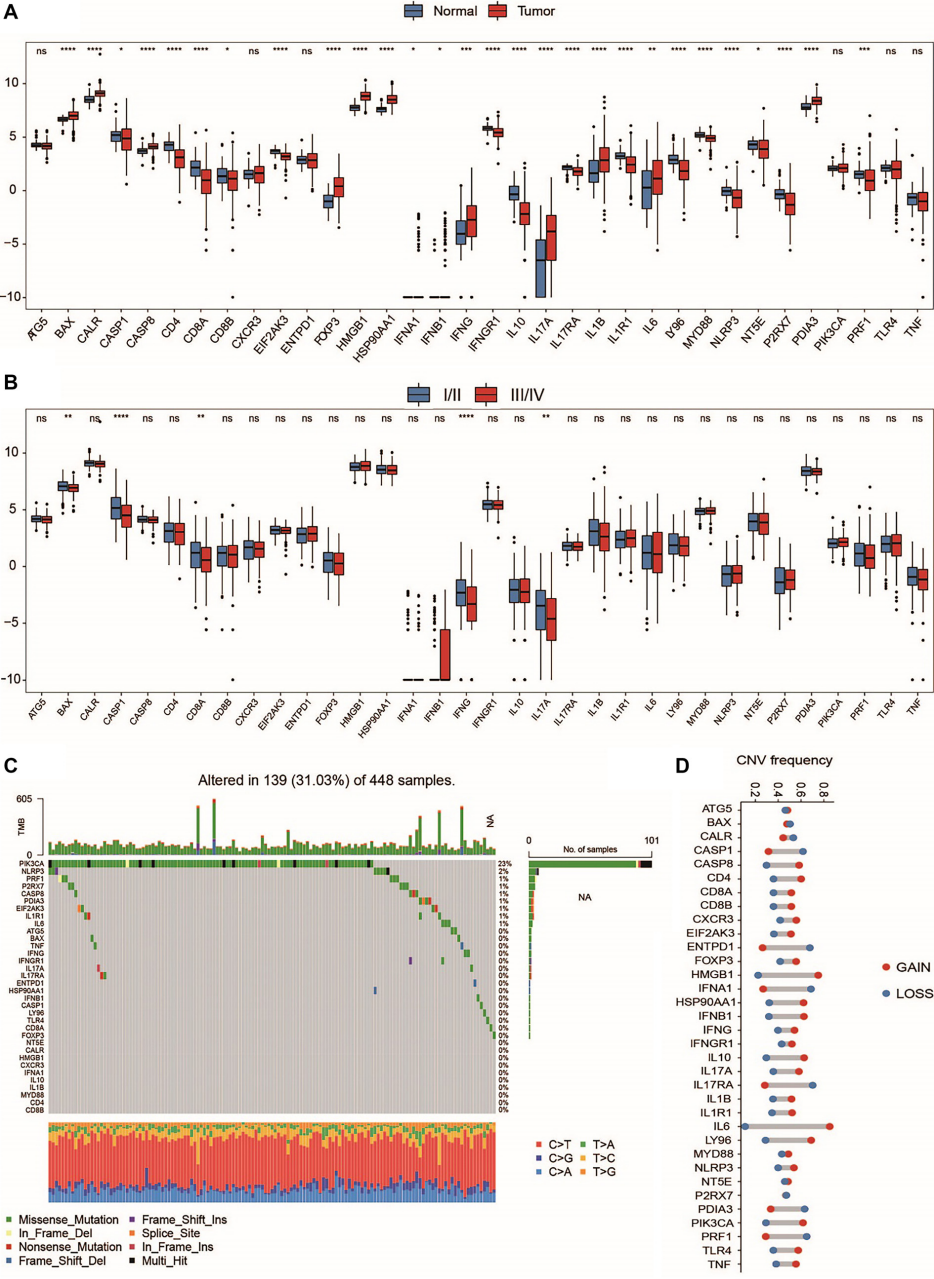
**Genetic landscape of ICD-related genes.** (A) Genes related to ICD between tumor and normal samples were subjected to differential expression analysis; (B) Between stage I/II and III/IV samples, differential expression analysis on the ICD-correlated genes with differences was performed; (C) Mutation landscape of ICD-correlated genes in the TCGA-CRC cohort; (D) CNV frequencies of ICD-correlated genes. ICD: Immunogenic cell death; TCGA: The Cancer Genome Atlas; CRC: Colorectal cancer; CNV: Copy number variation.

A total of 34 genes involved in the ICD process were selected for further investigation based on a recent study [22]. Differential expression analysis revealed that 82% (28 out of 34) of these genes were significantly altered in tumor tissues compared to normal samples in the TCGA-CRC cohort (Figure 1A). Notable examples include *CD8A*, *CD8B*, *CASP8*, *CASP1*, *CALR*, *HSP90AA1*, *IFNG*, *IFNGR1*, *PRF1*, *PIK3CA*, and *TNF*. CRC patients were stratified by TNM stage, lymphatic invasion, age, gender, and the presence of perineural invasion. Gene expression comparisons showed that *CD8A*, *CASP1*, *IFNG*, and *IL17A* were significantly downregulated in stage III/IV patients compared to those in stage I/II, suggesting a diminished immune response in more advanced tumors (Figure 1B). Additionally, significant differences in the expression levels of *CASP1*, *ATG5*, *EIF2AK3*, *ENTPD*, and *IL17A* were observed between patients with and without lymphatic invasion (Figure S1A). Moreover, expression of *ENTPD1*, *IL1R1*, *LY96*, *MYD88*, and *NLRP3* was significantly upregulated in patients with perineural invasion compared to those without (Figure S1B). In contrast, only a few genes were associated with age and gender in CRC (Figure S1C and S1D). These findings are critical for understanding immunomodulation in CRC and for guiding the development of targeted therapeutic strategies.

The genomic variation landscape of ICD-correlated genes was also analyzed. Overall, these genes exhibited a generally low mutation frequency, with the notable exception of PIK3CA, which displayed missense mutations in 23% of CRC samples (Figure 1C). This finding suggests that PIK3CA may contribute to immune evasion and influence treatment response in CRC. Additionally, copy number amplification of IL6 may upregulate the expression of pro-inflammatory factors, thereby promoting tumor progression and affecting the response to immunotherapy (Figure 1D). The observed variation patterns in these genes indicate that ICD-related genes may play a pivotal role in cancer immunomodulation and could serve as valuable biomarkers for predicting responses to immunotherapy.

### Screening ICD-related gene modules based on WGCNA

**Figure 2.**
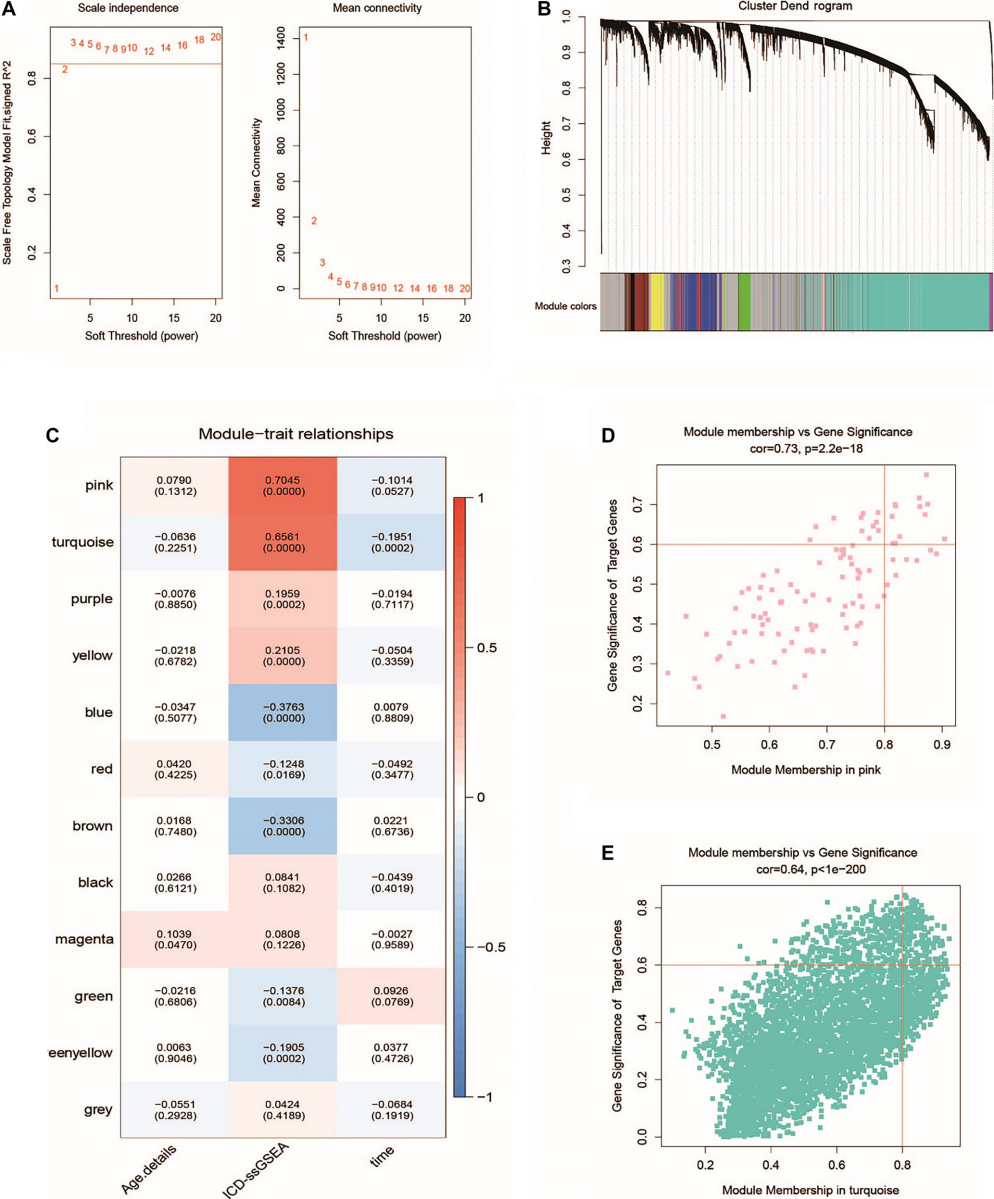
**ICD-related key gene screening.** (A) Scale-free fit index analyses of network topologies for various soft-thresholding powers. (B) Gene clustering dendrogram based on topological overlaps. Various modules were assigned different colors. (C) Module and clinical trait correlation study. MM and GS correlation analysis. Correlation analysis using scatter plots of the pink and (D) Turquoise modules (E). ssGSEA: Single-sample Gene Set Enrichment Analysis; ICD: Immunogenic cell death.

WGCNA was conducted to identify key ICD-related gene clusters. After removing outlier data, a soft threshold (β ═ 3, scale-free R^2^ ═ 0.850) was applied to ensure the network conformed to a scale-free topology (Figure 2A; Figure S2A). Subsequently, correlations between module eigengenes and ICD scores in CRC samples were calculated using ssGSEA (Figure 2B and 2C). The pink and turquoise modules, which showed stronger correlations than other modules, were selected for further analysis. Applying thresholds of cor.GS > 0.6 and MM > 0.8, a total of 183 ICD-associated hub genes were identified (Figure 2D and 2E). Protein–protein interaction (PPI) network analysis was performed using Metascape to further explore the interactions among these genes (Figure S2B). Functional enrichment analysis of GO biological processes revealed predominant enrichment in T cell activation, immune response-regulating signaling, immune response activation, and leukocyte proliferation (Figure S2C). These findings were consistent with the enriched cellular component and molecular function terms identified in the GO analysis (Figure S2D and S2E). KEGG pathway enrichment analysis further showed that these genes were involved in immune-related pathways, such as chemokine signaling and leukocyte transendothelial migration (Figure S2F).

### Development of the prognostic signature ICDRS for CRC

**Figure 3.**
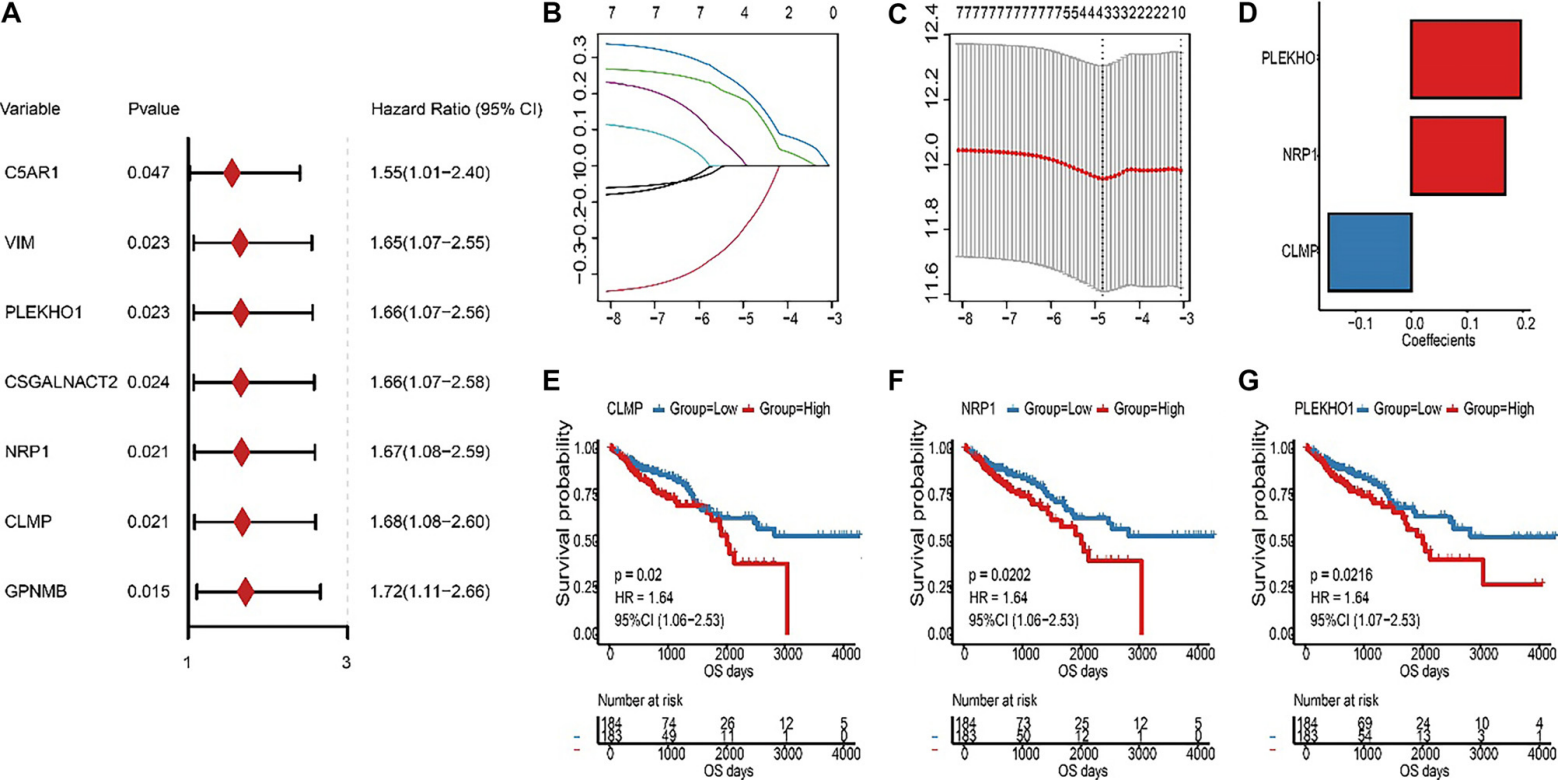
**ICD-related genes with prognostic significance.** (A) The univariate Cox regression analysis of ICD-related genes was presented as forest plot; (B) LASSO regression complexity controlled by the lambda; (C) LASSO regression confidence intervals of λ; (D) LASSO regression coefficients of the three key prognostic genes; (E–G) According to the expressions of key prognostic genes, the OS in low and high expression groups was visually compared according to Kaplan–Meier curves. ICD: Immunogenic cell death; OS: Overall survival; NRP1: Neuropilin-1.

To further identify key prognostic markers, we first selected seven ICD-related genes—*C5AR1*, *VIM*, *PLEKHO1*, *CSGALNACT2*, *NRP1*, *CLMP*, and *GPNMB*—using univariate Cox regression analysis (Figure 3A). We then performed LASSO Cox regression analysis to determine the optimal penalty parameter λ (Figure 3B and 3C), which was subsequently applied to the ICDRS model. Ultimately, three prognostic genes—*CLMP*, *NRP1*, and *PLEKHO1*—were selected to construct the ICDRS model (Figure 3D). Based on the median expression levels of these three genes, patients were categorized into high and low expression groups. As shown in Figure 3E–3G, all three genes were significantly associated with the prognosis of CRC patients.

### Evaluation and validation of ICDRS

**Figure 4.**
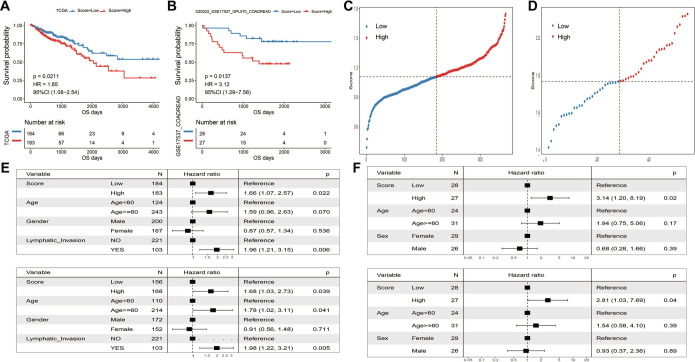
**Evaluation and validation of ICDRS.** (A) Kaplan–Meier curves of OS between the low-risk and high-risk groups based on the median ICDRS in the TCGA-CRC cohort; (B) According to the median ICDRS value in the validation cohort, Kaplan–Meier curves of OS were plotted for the two risk groups; (C) Risk score distribution in the TCGA-CRC cohort; (D) Risk score distribution in the validation cohort; (E) Univariate and multivariate Cox regression analyses to calculate risk score for TCGA-CRC patients; (F) Using univariate and multivariate Cox regression analyses for assessing the risk scores in validation cohort. ICDRS: Immunogenic cell death-related risk score; TCGA: The Cancer Genome Atlas; CRC: Colorectal cancer; OS: Overall survival.

ICDRS scores were calculated and evaluated in both the training and validation cohorts. Patients with high-risk scores had significantly poorer survival outcomes compared to those with low-risk scores (Figure 4A and 4B; log-rank test, *P* < 0.05). Moreover, neither cohort exhibited any extreme or abnormal events in the distribution of risk scores (Figure 4C and 4D). Univariate and multivariate Cox regression analyses confirmed that ICDRS serves as an independent prognostic indicator for the overall survival (OS) of CRC patients (Figure 4E and 4F). Collectively, these results suggest that the ICDRS signature may represent a novel prognostic biomarker for CRC.

**Figure 5.**
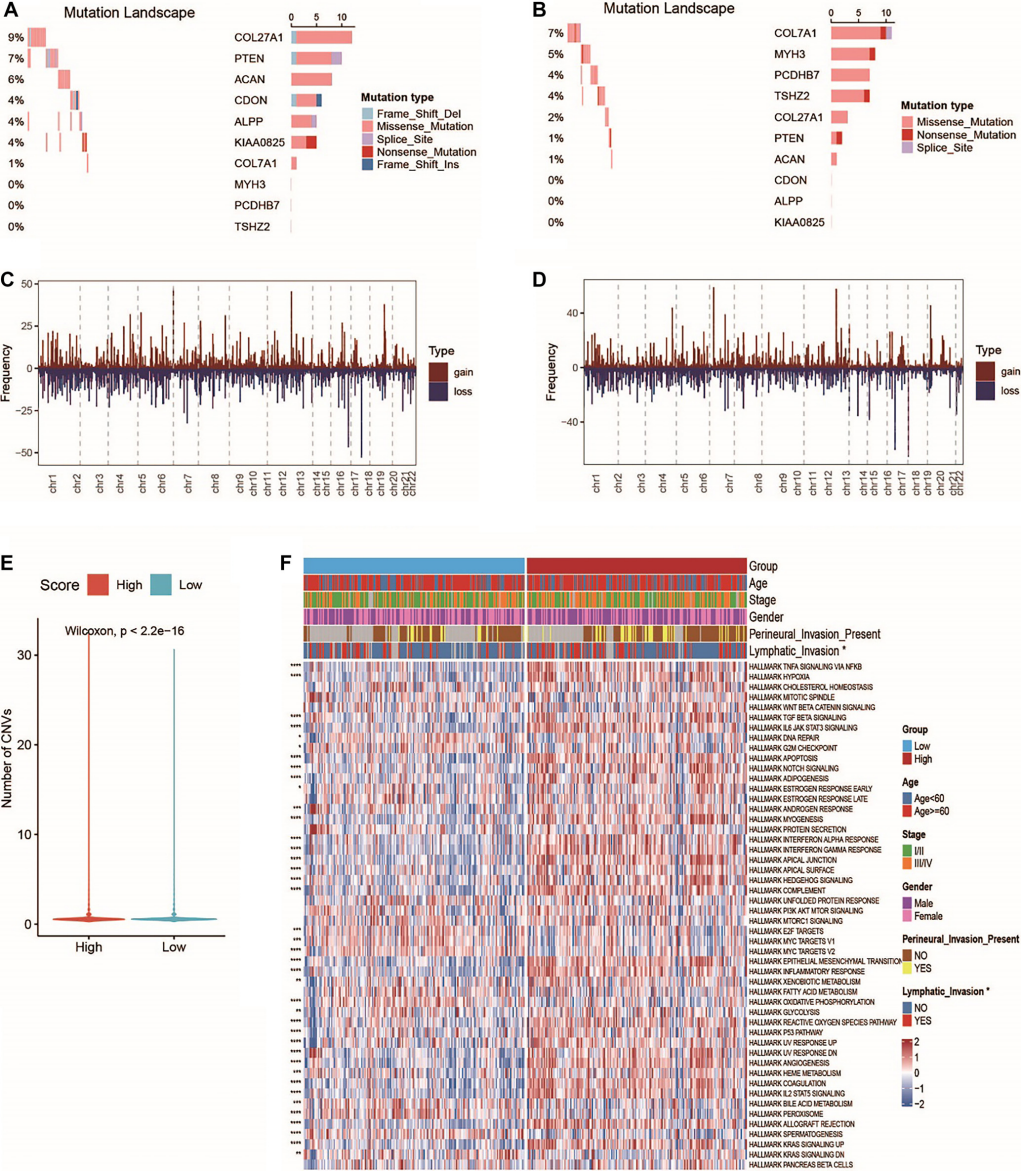
**Association between the ICDRS signature and molecular traits.** (A) The 10 most frequently mutated genes in the high-risk group were displayed in an oncoplot; (B) The 10 most frequently mutated genes in the low-risk group were displayed in an oncoplot; (C) Variations in copy numbers in the high-risk group; (D) Copy number variations in the low-risk group; (E) The distribution of copy number variations between the two risk groups; (F) Heatmap of the 50 signature pathway activity scores between the two risk groups. CNV: Copy number variation; ICDRS: Immunogenic cell death-related risk score.

### ICDRS revealed the molecular characteristics and pathway alterations in CRC

To explore the functional differences and molecular characteristics associated with ICDRS, patients were stratified into low-risk and high-risk groups based on their ICDRS scores. Compared to the low-risk group, high-risk patients exhibited higher mutation frequencies in *COL27A1* (9% vs 2%) and *PTEN* (7% vs 1%), specifically in the form of single nucleotide variants (SNVs). Conversely, mutations in *COL7A1* were more common in the low-risk group than in the high-risk group (7% vs 1%). Notably, the majority of these mutations were missense variants (Figure 5A and 5B). Additionally, the high-risk group showed significant gene amplifications and deletions across several chromosomal regions, while the low-risk group exhibited a generally lower frequency of CNVs (mean CNV value: 0.68 for low-risk vs 0.70 for high-risk; Figure 5C–5E, Table S2). To further assess functional differences, we evaluated the activity of 50 cancer hallmark signatures in the TCGA-CRC cohort. Substantial differences in hallmark pathway activity were observed between the two groups (Figure 5F). The high-risk group demonstrated elevated activity in pathways, such as HYPOXIA, TGF-β signaling, APOPTOSIS, NOTCH signaling, and the INTERFERON GAMMA RESPONSE. In contrast, the low-risk group showed upregulation of MYC targets and PEROXISOME-related processes. These findings support the utility of ICDRS in accurately distinguishing CRC patients based on distinct biological processes.

### Immune infiltration profiles defined by ICDRS

**Figure 6.**
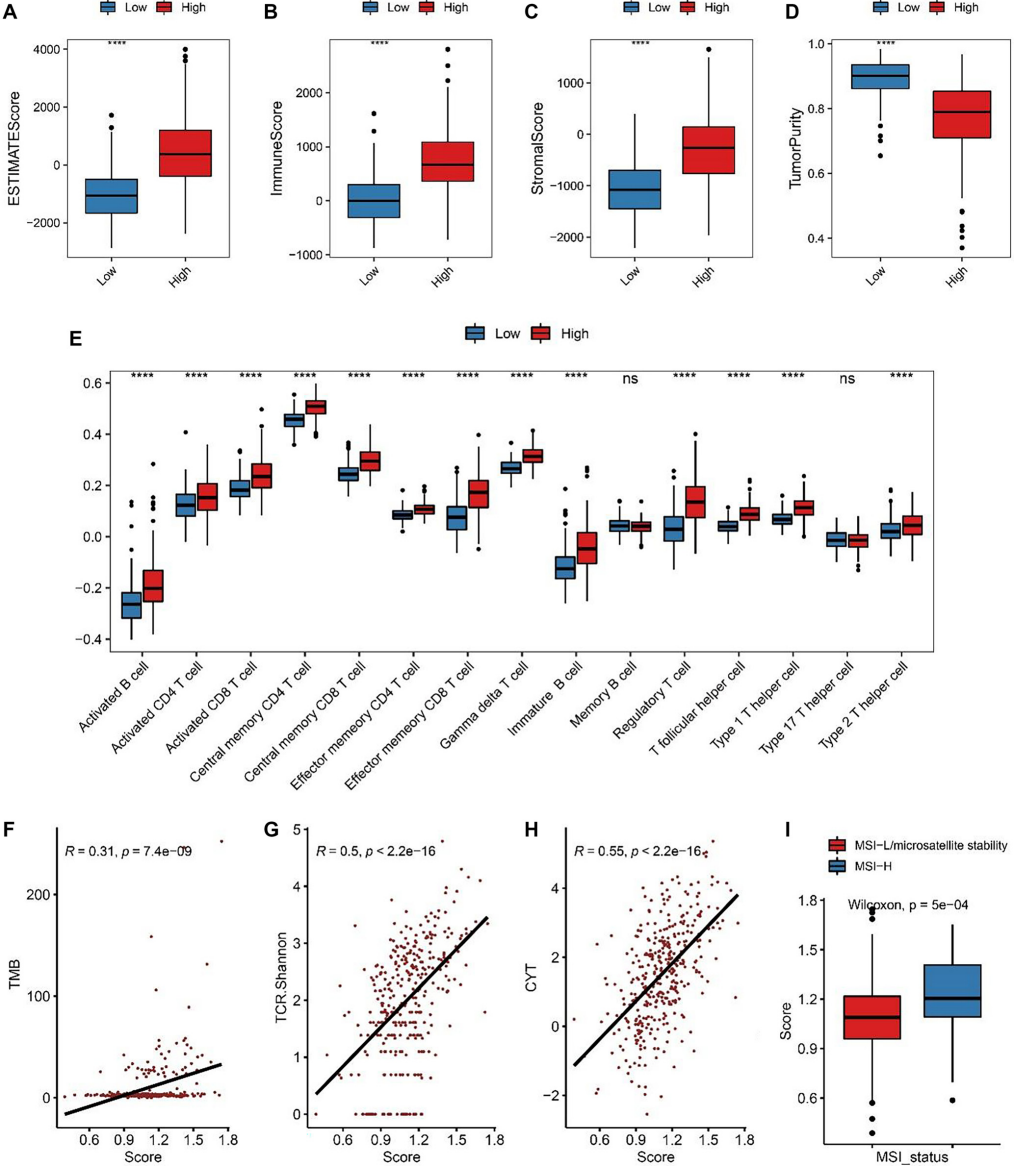
**The tumor immune microenvironment and immunogenomic characteristics of CRC related to theICDRS.** ESTIMATE score comparison (A), immune score (B), stromal score (C), and tumor purity (D) calculated using ESTIMATE between the high- and low-risk groups. (E) Comparison of the immune cell abundances between the two risk groups. Spearman correlation between the ICDRS risk score and tumor mutation burden (F), TCR diversity (G), and cytolytic activity (H). (I) ICDRS risk score distribution in the MSI-high and MSI-stability cohorts. To determine significance, the Wilcoxon rank-sum test was utilized. “ns”: *P* values > 0.05, “*”: *P* values < 0.05, “**”: *P* values < 0.01, “***”: *P* values < 0.001, and “****”: *P* values < 0.0001. TCR: T cell receptor; ICDRS: Immunogenic cell death-related risk score; CRC: Colorectal cancer.

ICDRS stratification was positively correlated with immune infiltration, as the high-risk group exhibited higher ESTIMATE, stromal, and immune scores—indicating greater immune cell infiltration and lower tumor purity (Figure 6A–6D). A comprehensive analysis of immune cell subtypes revealed elevated levels of immunosuppressive cells, such as T follicular helper cells and regulatory T cells, in the high-risk group (Figure 6E) [34, 35]. Additionally, the ICDRS was strongly associated with tumor mutation burden (Figure 6F, *R* ═ 0.31, *P* ═ 7.4e-09), T cell receptor (TCR) diversity (Figure 6G, *R* ═ 0.5, *P* < 2.2e-16), and cytolytic activity (Figure 6H, *R* ═ 0.55, *P* < 2.2e-16). The ICDRS was also significantly higher in the MSI-high group (Figure 6I, Wilcoxon rank-sum test, *P* ═ 5e-04). These findings suggest a complex TME in CRC, where immune suppression and anticancer immune responses coexist.

### ICDRS-guided chemotherapy strategies

**Figure 7.**
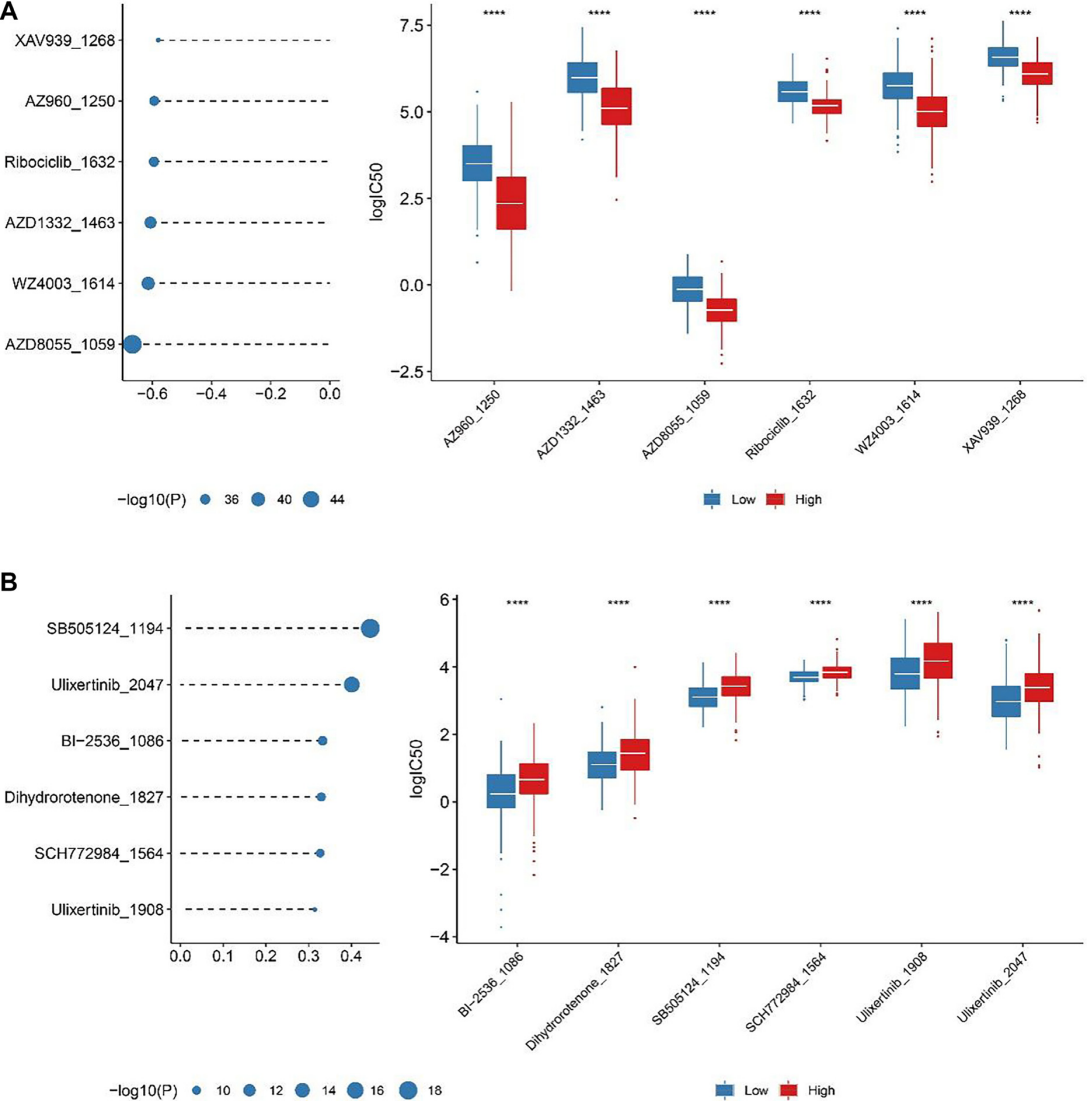
**Correlation of the sensitivity of drugs with ICDRS signature.** (A) Top six agents negatively associated with ICDRS; (B) Top six agents positive associated with ICDRS. ICDRS: Immunogenic cell death-related risk score.

By stimulating ICD with specific chemotherapy agents, tumors may become more susceptible to checkpoint blockade therapies. However, identifying the optimal combination of chemotherapy and immunotherapy remains a significant challenge [36, 37]. Since the ICDRS was developed based on ICD-associated genes, we hypothesized that it might also be correlated with chemotherapy response. The oncoPredict R package was used to estimate the IC50 values of various drugs. Spearman correlation analysis was then performed between the log2-transformed IC50 values of each drug and the ICDRS. The ICDRS was negatively correlated with sensitivity to AZ960_1250, AZD1332_1464, AZD8055_1059, ribociclib_1632, WZ4003_1614, and XAV939_1268 (Figure 7A). Notably, AZ960—a novel Jak2 inhibitor—has been reported to effectively induce apoptosis in cancer cells [38]. In contrast, the sensitivity to BI-2536_1086, dihydrorotenone_1827, SB5051_1194, SCH772984_1564, ulixertinib_1908, and ulixertinib_2047 showed a positive correlation with the ICDRS (Figure 7B), suggesting their potential as candidate treatments for cancer patients with varying ICDRS. Nevertheless, further research is needed to validate the association between ICDRS and drug susceptibility.

### The expressions of characterized genes in CRC cells

**Figure 8.**
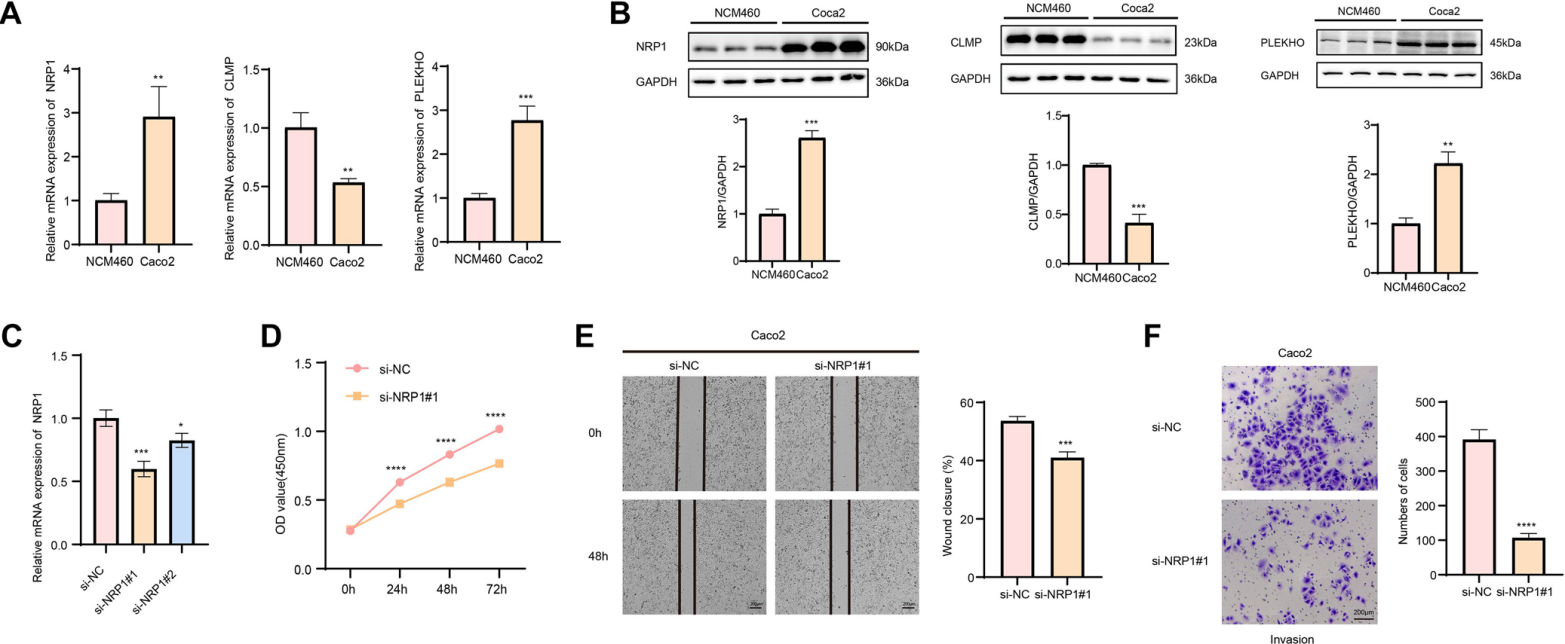
**The role of ICDRS signature on the biological function of CRC cells.** (A) The mRNA expression levels of *NRP1*, *CLMP*, and *PLEKHO* in NCM460 and Caco2 cells, respectively; (B) The protein expression levels of *NRP1*, *CLMP*, and *PLEKHO* in NCM460 and Caco2 cells, respectively; (C) Based on qRT-PCR to verify the efficiency of *NRP1* knockdown (si-*NRP1*#1 and si-*NRP1*#2); (D) CCK-8 assay to verify the effect of *NRP1* knockdown on the proliferative capacity of CRC cells; (E) Wound healing assay to assess the effect of *NRP1* on the migration of CRC cells; (F) Transwell assay to assess the ability of *NRP1* knockdown to inhibit invasion of CRC cells. All procedures were subjected to three independent repetitive tests. “*”: *P* values < 0.05, “**”: *P* values < 0.01, “***”: *P* values < 0.001, and “****”: *P* values < 0.0001. NRP1: Neuropilin-1; ICDRS: Immunogenic cell death-related risk score; qRT-PCR: Quantitative reverse transcriptase PCR; CRC: Colorectal cancer.

To further validate the prognostic signatures we identified, we first examined the expression levels of *CLMP*, *PLEKHO*, and *NRP1* in CRC cells (Caco2) and normal colonic mucosal cells (NCM460) using qRT-PCR and Western blotting. The mRNA expression levels of *PLEKHO* and *NRP1* were significantly elevated in CRC cells compared to NCM460 cells, while *CLMP* expression was significantly downregulated (Figure 8A). Consistently, the protein levels of these genes mirrored the mRNA expression patterns (Figure 8B). Previous studies have shown that *NRP1* is closely associated with tumor progression and metastasis and is significantly linked to poorer patient survival in CRC [39]. Based on this evidence, we selected *NRP1* for further investigation to evaluate the impact of its knockdown on CRC cell proliferation, migration, and invasion (Figure 8C). CCK-8 assay results demonstrated that silencing *NRP1* expression significantly suppressed the proliferation of CRC cells (Figure 8D). Additionally, *NRP1* knockdown markedly inhibited CRC cell migration and invasion (Figure 8E and 8F). These findings suggest that prognostic markers identified based on ICD-related genes may play important roles in the development and progression of CRC.

## Discussion

Advancements in treatment have been made; however, CRC remains a deadly disease characterized by significant heterogeneity. This variability underscores the need to optimize therapies to improve survival rates and reduce mortality. As such, identifying reliable prognostic biomarkers is essential for stratifying survival risk and guiding therapeutic strategies tailored to specific subtypes. Li et al. employed a multistep approach to construct a signature map based on immune-related genes using data from the TCGA and GEO databases. Their findings indicated that CRC patients with low immune risk scores experienced better outcomes with immunotherapy [40]. Similarly, Zhao et al. [41] explored the molecular characteristics of PANoptosis in CRC prognosis and developed a predictive model incorporating four PANoptosis-related genes: *TIMP1*, *CDKN2A*, *CAMK2B*, and *TLR3*. The ICDRS offers a distinct advantage over existing prognostic indicators in assessing CRC patient outcomes. As a novel form of regulated cell death, ICD has been shown to enhance adaptive immunity and amplify anti-tumor immune responses. This suggests that identifying ICD-related biomarkers could help pinpoint CRC patients more likely to benefit from immunotherapy [42]. The ICDRS, based on the expression of ICD-related genes, captures complex changes within the TME. It enables more accurate identification of high-risk patients who may require intensified treatment or immunotherapy, offering greater predictive accuracy and enhanced value for individualized treatment planning.

In this study, we first assessed the expression differences of ICD-correlated genes in both CRC and adjacent normal tissue samples using public databases, and analyzed the variants of ICD-related genes in the TCGA-CRC cohort. The intracellular mediator phosphatidylinositol-3-kinase (*PI3K*), encoded by the *PIK3CA* gene, plays a crucial role in promoting cell transformation and proliferation, tumor initiation, and resistance to apoptosis. Activation of *PI3K* occurs in response to external growth factors and hormones [43]. Dysregulation of *PI3K* leads to the activation of AKT, a serine/threonine kinase, in various cancer types, ultimately affecting numerous downstream proteins that drive unchecked cellular and tumor proliferation [44]. Approximately 15%–20% of CRC cases harbor activating mutations in *PIK3CA*, which are associated with OS and progression-free survival in CRC patients [45]. Moreover, *PIK3CA* mutations are linked to distinct immune profiles in gastric cancer and can modulate tumor immunogenicity [46]. Notably, we observed a high mutation frequency of *PIK3CA* in CRC samples based on the mutation profiles of ICD-associated genes, suggesting that *PIK3CA* mutations may influence CRC growth and progression via DAMPs, by altering the tumor’s immune response.

The ICDRS was developed for CRC by integrating LASSO Cox regression analysis, univariate Cox regression, and WGCNA. It demonstrated strong predictive power for independently assessing the survival outcomes of CRC patients. The robustness of the signature was validated using both internal and multiple external datasets. Notably, many genes analyzed in this study have previously been associated with CRC. For example, *C5AR1* acts as a master regulator in CRC tumorigenesis through immune modulation [47]. The expression of VIM changes in Caco2 cells after co-cultivation with CRC-associated bacteria [48]. The prognostic relevance and underlying mechanism of *PLEKHO1* in the immune microenvironment of colon cancer have also been reported [49]. *PLEKHO1* contributes to the development of renal cell carcinoma, and its knockdown significantly inhibits cancer cell viability while promoting apoptosis [50]. CLMP regulates colon epithelial cell proliferation and helps prevent tumor growth [51]. It also has an anti-CRC effect and influences the resistance of CRC cells to all-trans retinoic acid [52]. *NRP1*, an important immunomodulatory receptor, is closely linked to CRC progression. Its role in the TME is multifaceted, involving both immunosuppression and angiogenesis [53]. *NRP1* suppresses anti-tumor immune responses by enhancing regulatory T cell infiltration and promoting immune escape [54]. It also activates angiogenic pathways by interacting with vascular endothelial growth factor receptor 2 (VEGFR2), thereby increasing nutrient supply to tumors and driving tumor growth and metastasis [55]. This study is the first to demonstrate the impact of *NRP1* on CRC cell proliferation, migration, and invasion based on ICD-related genes. Thus, *NRP1* is not only a key mediator of tumor immunomodulation but also plays a central role in angiogenesis. Targeting *NRP1* may help restore immune responses and inhibit angiogenesis, offering a promising strategy for both immunotherapy and anti-tumor treatment.

CRC is often associated with chronic inflammation [56]. Inflammation in the gastrointestinal tract can trigger cancer-promoting genetic changes and initiate CRC development. Additionally, immune cells, such as myeloid and lymphoid cells infiltrate tumors and drive “tumor-provoked inflammation,” which promotes cancer progression by supporting the survival and proliferation of malignant cells [57, 58]. In this study, we identified two ICDRS subtypes with distinct TME profiles. A higher ICDRS was associated with increased infiltration of various immune cells, suggesting the coexistence of both pro- and anti-tumor components within the TME. The presence of activated CD4+ and CD8+ T cells in CRC patients has been closely linked to effective antitumor immunity [59, 60], while follicular helper T cells are also associated with improved survival outcomes in CRC [61]. On the other hand, Th17-type cytokines can promote CRC tumorigenesis by activating the *STAT3* and NF-κB pathways [62]. Given this dual nature of immune activation and suppression, GSVA revealed that immune-related characteristics were enriched in the high ICDRS group. Compared to patients with low ICDRS scores, those with high ICDRS are more likely to benefit from checkpoint inhibitor therapy, as they exhibit elevated levels of immune checkpoints.

### Limitations

Despite these promising findings, the present study has some limitations. The analysis of the relationship between ICDRS and therapeutic sensitivity to anti-PD-L1 treatment was constrained by the limited availability of data from CRC patients undergoing immune checkpoint blockade (ICB) therapy. To gain a deeper understanding of the molecular mechanisms underlying CRC immunobiology, future research should aim to validate the prognostic value of ICDRS using larger, multi-omics datasets. Additionally, transcriptomic analyses could be further enhanced by integrating proteomic and metabolomic data. Importantly, the functional roles of the identified prognostic genes in CRC should be validated through experimental studies, such as mouse xenograft or gene knockout models.

## Conclusion

This study established and validated a robust ICD-correlated prognostic signature that accurately predicts survival outcomes and reveals distinct immune profiles and molecular characteristics between the two CRC risk groups. Further research and validation are needed to explore the therapeutic implications of this signature in CRC.

## Supplemental data

Supplementary data are available at the following links:

https://www.bjbms.org/ojs/index.php/bjbms/article/view/12028/3857

https://www.bjbms.org/ojs/index.php/bjbms/article/view/12028/3834

https://www.bjbms.org/ojs/index.php/bjbms/article/view/12028/3835

## Data Availability

The datasets analyzed herein are publicly available at the Gene Expression Omnibus (GSE17537) (https://www.ncbi.nlm.nih.gov/geo/GSE17537) and TCGA (https://portal.gdc.cancer.gov/) databases.
